# Aloe Socotrina, an Anti-psoric Remedy

**Published:** 1888-11

**Authors:** W. P. Wesselhœft

**Affiliations:** Boston


### ALOE SOCOTRINA, AN ANTI-PSORIC REMEDY.

W. P. WESSELHŒFT, M. D., BOSTON.

Dr. Hering says, "Aloe has many symptoms like Sulphur and is equally important in chronic diseases with abdominal plethora," etc.

Dr. Whiting, of Danvers, Mass., related to me the cure of a case of chronic diarrhœa caused by the suppression of an eczema years before. The cure was effected in a few weeks and patient remained well *after the reappearance of the old eczema,* which followed in a few weeks after the dose of Aloe was administered.

This remedy *alone* without the necessity of following it with a so-called anti-psoric, has proved curative in my hands in old intestinal and hemorrhoidal affections, which in several instances were followed by the reappearance of cutaneous diseases known to have been suppressed by external means. In Dr. Whiting's case the patient had an itch suppressed in childhood which reappeared with great violence and a magic cure of the diarrhœa followed. I recall an instance of hemorrhoids which were greatly relieved by the application of cold water, the burning in them being especially aggravated after a stool. Aloe had been given, and several weeks after the hemorrhoids ceased burning an itching eruption appeared upon the interior surface of both thighs, which was especially annoying after getting warm in bed. The temptation to give Sulphur was very great, but as the general condition of the patient improved this temptation was resisted and the eruption after several months passed away and the hemorrhoids remained without pain or burning, giving him no annoyance.

When such experiences repeat themselves in the treatment of chronic diseases, I think it no hasty assumption to give this drug a place among such heroes of our *Materia Medica* as Sulphur,

Calcarea, Lycopodium, etc. In giving it such a position I claim for it a proper respect and allowing its action to fully expand itself before another dose is given, or its action carelessly interferred with by another remedy. If we are confident of its powerful centrifugal action, and know that it can reproduce on the surface cutaneous diseases long before suppressed, or has the capacity of bringing to the surface latent psoric tendencies, which may have caused internal discord through a lifetime, affecting different systems and different tissues at different periods of a lifetime, disturbing the harmony of the vital force in the most varied expressions, then, I say, we may safely trust it to do still more and annihilate even the product it has caused to appear.

The action of Aloe is a very long one. I am not prepared to say how long, but I am certain that it may continue curative for three months, and possibly much longer.

The pathogenesis of Aloe, as we now possess it in the *Guiding Symptoms*, is one of those masterpieces of Constantine Hering's genius, which he repeated in his Nux moschata, Stramonium, etc. He arranged and collected Helbig's and Buchner's provings and added all clinical results up to the year 1881. For this we owe him a great debt of gratitude, which we can best pay his memory by making the right use of his labor, and strictly individualizing our cases, never prescribing on pathological indications only. With his marvelous insight into the sphere of the action of a drug he has bequeathed the strongest characteristics for the use of Aloe, and it is our fault if we neglect to profit by such masterly achievement.

The following case will demonstrate the thorough work which this drug is capable of performing in a single, highly potentized dose.

Mrs. F. H. M., aged thirty-six, light blonde, medium size, thin, pale.

Has suffered with gastralgia and diarrhœa for four years.

Awakes almost every night at two A. M. with oppression at stomach, nausea, saliva running copiously from the mouth. After violent rubbing over epigastrium, she raises gas, which gives relief. There is usually much rumbling of gas in abdomen with these attacks, but does not feel secure in making the effort to pass it, as she fears stool will escape.

She is called out early in morning, sometimes before daylight, by urgent desire for stool.

The stool is painless, watery, gushes out with flatus.

Before stool rumbling in abdomen, or as if fluid was swashing.

This condition of things occasionally alternates with constipation of several days' duration, when one or two formed stools may occur, to be surely followed by a longer attack of diarrhœa.

The stools always occur in early morning and during forenoon; seldom has more than four stools daily, which are followed by some exhaustion.

Tongue slightly coated yellow. Appetite usually good.

After eating has had for several years a dull pain under right shoulder-blade, lasting from half to one hour.

Seven years ago a moist eruption appeared on chin and submaxillary region, exuding a thin, clear serum with intense itching; scratching aggravated the moisture and itching. This was treated locally with Zinc ointment and other applications and finally "cured." Soon after it (she does not now remember how long, but probably several months) her stomach began to give her trouble, and the pain under the right shoulder-blade after eating disappeared. Later the diarrhœa followed, alternating with constipation.

Has had two children, the youngest five years old.

Is taking pills of Opium and a mineral acid (probably Sulphuric).

As she had taken these drugs up to the time of coming to me, I gave her a dose of Nux vomica$^{cm}$.

She returned in a week to tell me that the nocturnal gastric attacks had been less severe and the stools somewhat less urgent and less fluid. I allowed her to go another week without medicine.

At the end of the second week after taking Nux vomica the report was: Stools equally loose, frequent, and urgent; do not appear quite as early but still drive her out of bed; the same sense of insecurity of sphincter when desiring to pass flatus. During the day occasional griping in abdomen independent of stool. Tongue a little cleaner. She now received a dose of Aloe$^{cm}$.

In a week she reported that during the last two or three days she had perceived an improvement in the urgency of the stool and had passed flatus several times without the feeling of insecurity. For the past three days she has had but two stools, less watery and gushing.

She now received during two months only Sac. lactis; the stools became normal with recurrence of the nocturnal gastralgia. At the end of the sixth week after taking Aloe a large stye appeared on the lower lid of the left eye. This healed and was followed a week later by a boil on the end of the nose, and an annoying soreness on the inside of the right nostril, which scabbed over, and after shedding the scab remained raw for a period, till a new scab formed. At the end of another month the nose had healed internally, and my patient has now been free from all stomach and bowel troubles for many months.

Aloe was selected only on account of its symptoms of the diarrhœa, which were very characteristic, and the selection therefore not a difficult one.

Aloe, however, as far as observed, has none of the gastric symptoms similar to this case, and still it cured the after midnight attacks of oppression, nausea, and salivation. The dull pain under the shoulder-blade after eating does not appear in the proving of this drug, yet this oldest of all her symptoms yielded to its action, although it was the last to disappear; not, however, until some time after the disappearance of the boils and eczematous disease in the nostril.

From this case we should draw the following lesson: Aloe will cure chronic diarrhœa when it is homœopathically indicated, clear away a number of other psoric symptoms not yet contained in its pathogenesis. It does not need to be repeated to fulfill its mission, and is capable of reproducing cutaneous disease after having been suppressed eight years.

The question now arises, are we authorized to add to the pathogenesis of Aloe those symptoms which were cured in this case, notably the pressure under right shoulder-blade after eating, and the oppression in the stomach, nausea, and salivation occurring at two A. M. and relieved by rubbing. I think we are fully authorized to do so and in this way enrich still further this splendid proving.

---

Dr. Hitchcock—Mr. President, I have a case of Aloe, and I would like to have a suggestion in relation to it. A patient came to me from my predecessor, who had been troubled for a number of years with hemorrhoids, and he had always received Aloe, which relieved. I found on my first prescription that Aloe was the remedy then, and he would receive relief for about three or four months, when it would return again, and Aloe repeated would relieve him. Now that has been going on for a long time, and I want to ask the question, Is Aloe the remedy to cure that? I have given nothing else. Is there anything else?

Dr. Allen—My experience with Aloe is that it is very similar to Lycopodium—a bad remedy with which to begin the treatment of a chronic case—and if I begin with it I must return to an anti-psoric to cure. Sulphur should usually precede Aloe in the treatment of these chronic cases and Lycopodium should frequently follow.

Dr. Sawyer—I have had some Aloe cases. I had one somewhat over two years ago—a lady about seventy. It was a clear case of Aloe diarrhœa. I gave one dose of the 100th. This was a case of many months' standing and very troublesome. There were but two or three passages afterward that were abnormal. She remained in good health for several months, when it returned again, and a single dose checked it. A single dose does check it; but it does recur at intervals of from three to four months.

Dr. Nash—The thought occurred to me while that paper was being read of what Hahnemann used to teach—that where we find a seemingly indicated remedy, removing the symptoms, and those symptoms returning again and again, it indicated that there was some constitutional taint at the bottom of the case that needed an anti-psoric remedy. Then the question occurred that if this remedy, Aloe, is an anti-psoric, we have yet an example, as Dr. Hitchcock relates, of the symptoms returning again and again. Anti-psorics are intended to remedy that. This certainly would be against Aloe being an anti-psoric. If not, after using an anti-psoric remedy and the symptom returns, what shall we return to then?—*Clinical Bureau, I. H. A.*, 1888.

